# Differences in Influencing Factors Between Non-suicidal Self-Injury and Suicide Attempts in Chinese Adolescents: The Role of Gender

**DOI:** 10.3389/fpsyt.2022.870864

**Published:** 2022-06-30

**Authors:** Huiqiong Xu, Zhicheng Jiang, Shuqin Li, Xinyu Zhang, Shaojun Xu, Yuhui Wan, Fangbiao Tao

**Affiliations:** ^1^Department of Maternal, Child and Adolescent Health, School of Public Health, Anhui Medical University, Hefei, China; ^2^Key Laboratory of Population Health Across Life Cycle, Ministry of Education of the People's Republic of China, Anhui Medical University, Hefei, China; ^3^NHC Key Laboratory of Study on Abnormal Gametes and Reproductive Tract, Hefei, China; ^4^Anhui Provincial Key Laboratory of Population Health and Aristogenics, Hefei, China

**Keywords:** non-suicidal self-injury, suicide attempts, psychological symptoms, childhood maltreatment, adolescents

## Abstract

**Introduction:**

Non-suicidal self-injury (NSSI) and suicide attempts (SA) are common in adolescents and are important risk factors for suicide deaths. They are related to various psychosocial, behavioral, and biological factors. We aimed to compare the differences on psychological behavior problem and family environment characteristics between NSSI and SA, and the role of gender.

**Methods:**

A multi-center population-based survey was conducted in 29 schools across 4 provinces in China. A total of 14,500 urban and rural adolescents in grades 7–12 completed a structured questionnaire to report their sociodemographic information, psychological and behavioral characteristics, childhood maltreatment, parent-child relationships, NSSI, and SA. *Post-hoc* tests, pairwise comparisons, and multinomial logistic regression analyses were conducted to explore the differences and similarities between subjects who had engaged in NSSI and SA.

**Results:**

The prevalence of NSSI and SA were 27.3 and 4.9%, respectively, and the co-occurrence of these two behaviors (NSSI + SA) was reported to 2.8%. The NSSI + SA group scored the highest on all study variables, followed by the SA-only group, the NSSI-only group, and the non-self-harm group (*p* < 0.001). Compared with the non-self-harm group, adolescents who reported either NSSI or SA scored significantly higher on all study variables (*p*< 0.0083). The comparison between other self-harm groups, this difference have varied in all research variables.

**Conclusion:**

The current study indicate that psychological, behavioral, and family relationships profiles of Chinese adolescents with SA and NSSI are similar, but the measured problematic characteristics were more severe in suicide attempters. In the future, it's necessary to pay more attention to adolescents with more serious psychological and behavioral problems to prevent and early intervene in their self-harm, and actively explore gender differences.

## Introduction

Non-suicidal self-injury (NSSI) and suicide attempts (SA) in adolescents are major public health concerns worldwide. NSSI is interpreted as deliberate, self-inflicted damage of body tissue without suicidal intent for purposes not socially or culturally sanctioned (1). SA refers to engagement in potentially self-injurious behavior with some intent to die from the behavior (2). Among adolescent community samples in many countries, the lifetime prevalence of NSSI ranges from 15.1 to 38.6% (3–5), while the lifetime prevalence of SA ranges from 3.2 to 7.4% (6, 7). Both NSSI and SA are associated with numerous negative life outcomes and have significant economic and social consequences.

The relationship between NSSI and SA is complicated. A comprehensive review summarized several studies that specifically examined the association between NSSI and suicidal behavior, proposed three theories, and introduced an integrated model to account for the link between NSSI and suicidal behaviors (8). Research shows that a prior history of NSSI is one of the risk factors and strongest predictors for SA both cross-sectionally and longitudinally (9–11). NSSI and SA can occur independently or simultaneously. Some scholars also believe that NSSI and SA have common risk factors, which may include behavioral problems caused by the “third variable” (8). Several studies have identified multiple psychosocial factors associated with SA and NSSI, including borderline personality disorder (12), polysubstance use (13), psychiatric disorders, psychological vulnerability, nightmares (14), hopelessness (15), impulsiveness (16), female gender, lower academic performance, depression, substance use (tobacco and alcohol), low self-esteem (17). Common neurobiological bases, including indices of serotonergic function (18), neuro-immunological biomarkers, the brain-derived neurotrophic factor and other neuromodulators (19), are also risk factors for both SA and NSSI. However, NSSI and SA have been differentiated by intention, frequency, age at onset, and lethality of behavior, and they have different psychosocial and epidemiological characteristics.

Based on their history of self-harm, adolescents can be divided into four groups: Non-self-harm (NSH), NSSI-only, SA-only, and NSSI+SA. Adolescents with different histories of self-harm may have different influencing factors. However, little is known about the differences and similarities of influencing factors between different types of self-harm in the general population of adolescents. To our knowledge, factors that distinguished the NSSI + SA group from the NSSI-only group are female gender, lower grade, impulsivity, health risk behaviors, and family cohesion (7). Compared with the NSH group, adolescents with either NSSI or SA have been shown to score significantly higher on trait anger, impulsiveness, hopelessness, internalizing, and externalizing problems (20). In addition, several studies have indicated that the family environment—parental attitudes, parent-child relationships, and childhood maltreatment—is more important and affects the development of the child (21–24). Emerging unhealthy eating habits, such as consuming fast foods (FFs) and sugar-sweetened beverages (SSBs), Internet addiction behaviors, and problematic mobile phone use (PMPU) also have a significant impact on the physical and mental health of adolescents (25–27). These factors are important to an individual's development. Therefore, it has also been suggested that the distribution of these variables may differ in self-harm groups. Hence, we sought to add to the extant literature by investigating these variables.

Moreover, epidemiological research on the prevalence of NSSI and SA has shown gender differences, with some reporting a higher prevalence among women than men, and other studies showing no differences (28, 29). However, few studies have investigated gender differences in the influencing factors of NSSI and SA. Therefore, the objective of this study was to illustrate the current differences in influencing factors between NSSI behaviors and SAs among a large population of Chinese adolescents aged 12 to 20 years and to explore whether the roles of those factors varied by gender status.

## Methods

### Sample and Procedures

We conducted this cross-sectional study from November 2017 to January 2018 with nationwide sample surveys in China. Participants were students in grades 7–12, selected using multistage stratified cluster random sampling. We first determined the sample area, considering geographic location, demographic composition and economic development in China, we included students from the southern (Shenzhen City in Guangdong Province. 3,465, 23.9%), central (Zhengzhou City in Henan Province. 3,678, 25.4%), eastern (Nanchang City in Jiangxi Province. 3,697, 25.5%), and western (Guiyang City in Guizhou Province. 3,660, 25.2%) regions. Also, Shenzhen and Zhengzhou are first-tier cities (7,143, 49.3%), while Nanchang and Guiyang are second-tier cities (7,357, 50.7%). Second, we randomly chose eight schools, junior and senior high, from each region (including four rural schools and four urban schools for each) and recruited general juniors and seniors as participants (excluding those from experimental or key schools). As three were combined junior and senior schools, a total of 29 schools were selected for inclusion. Third, we selected three classes from each grade as investigation samples. If the three classes were not enough for the sample size, we randomly sampled adjacent classes until the sample size was sufficient. In addition, this study population was recruited from the National Adolescent Health Surveillance Study, which has been conducted at 1-year intervals, all regional cooperative units have good compliance, thus facilitating the data collection.

The design and data collection were approved by the Ethics Committee of Anhui Medical University (ref. no.: 20170290). All study procedures were conducted in accordance with the Declaration of Helsinki. Informed consent was obtained from the students and the parents or guardians.

### Measures

#### Sociodemographic Variables

The surveys included self-reported information on sociodemographic characteristics including age, gender (male/female), grade (middle school or high school), single child status, boarding school, urban/rural residency, educational level of father and mother (less than junior middle school, junior middle school, senior middle school, college or more). We also asked students about the self-perceived economic status of their family (poor, moderate, or good), and how many close friends they have (none, 1–2, 3–5, or ≥ 6).

#### Non-suicidal Self-Injury

The Adolescent Non-suicidal Self-injury Assessment Questionnaire, which included 12 items, was also used in this study (30). Participants answered yes or no to each of the NSSI methods: pinched or scratched yourself, banged your head or fist against something hard (e.g., a wall, a tree, etc.), hit yourself with your fists or palms, pulled your own hair on purpose, bitten yourself, cut or pierced yourself, burn yourself intentionally (as with a cigarette end, boiling water, lighter, or match), engraved words or symbols on the skin intentionally. For those who admitted that they had engaged in a certain behavior of NSSI, the frequency of NSSI was asked. The total number of occurrences of all NSSI items was calculated as the total frequency of NSSI; students with an NSSI frequency > “1” were considered to have “engaged in NSSI.” Cronbach's α for NSSI in the present study was 0.919.

#### Suicide Attempt

According to the definitions of attempted suicide in the American Youth Risk Behavior Surveillance System (YRBSS) (6) and previous studies by our research group (5), this study mainly examined the occurrence of suicide attempts in the last year. The question given was, “How many times have you tried to kill yourself in the past 12 months?” and was answered as one of the following four options: 0 times, 1 time, 2–3 times, or ≥4 times. An answer indicating one or more attempts was considered SA.

#### Psychological Symptoms

The psychological symptoms experienced by the student in the preceding 3 months were assessed using the Multi-dimensional Sub-health Questionnaire of Adolescents (MSQA) (31, 32). The scale includes a total of 39 items in the three dimensions of emotional problems (EPs), conduct problems (CPs) and social adaptation problems (SAPs). EPs are measured using 17 questions (e.g., Do you always feel nervous?). CPs are measured using nine questions (e.g., Do you have the impulse to damage something?). SAPs are measured using 13 questions (e.g., Can you quickly adapt to new learning environments?). Each question is answered from 1 (lasts for more than 3 months) to 6: (no or last <1 week). Total scores of each participant's items were calculated; the higher the score, the longer the symptoms lasted, and the more serious the psychological symptoms. The MSQA has been shown to have high reliability and validity; Cronbach's α for the MSQA was 0.968 in this study.

#### Childhood Maltreatment

Childhood maltreatment was evaluated using the Childhood Trauma Questionnaire (CTQ) (33), which was localized by Zhao et al. (34). The questionnaire includes five subscales of emotional abuse, physical abuse, sexual abuse, emotional neglect, and physical neglect, each with five questions. All participants were asked, “While you were growing up (during your first 18 years of life), how often did someone do any of these things to you?—No one at home cared about my hunger, someone in my family called me ‘stupid,’ ‘lazy’ or ‘ugly,’ I was taken care of and protected” and so on. Each item is scored on a Likert scale from 1 (never) to 5 (very often). Part some items are reverse-scored. Possible scores range from 5 to 25 for each abuse subscale; total scores range from 25 to 125. The Cronbach's α for the CTQ in this study was 0.748.

#### Parent-Child Relationship

The parent-child relationship scale is FACES III (Family Adaption and Cohesion Evaluation Scales III) subscales are based on Olson et al. (35) and others in FACES II as proposed in 1985. The Chinese version of the parent-child relationship scale was translated and revised by Zhang et al. (36), which consisted of 10 items. The two subscales of father-child relationship (FCR) and mother-child relationship (MCR) were exactly the same with a modified response format from 1 (almost always) to 5 (little); questions 3, 4, 8, and 9 were reverse-scored. The scale was interpreted as the higher the score, the worse the parent-child relationship status. The Cronbach's α coefficient was 0.85 (father) and 0.90 (mother), respectively.

#### Problematic Mobile Phone Use

We used the Self-Rated Questionnaire for Adolescent Problematic Mobile Phone Use (SQAPMPU), designed by Tao et al. (37) for use in adolescents, to evaluate PMPU. It contains 13 items in three dimensions: withdrawal symptoms, craving, and physical and mental health status. For instance, “I don't feel like I spend enough time on my phone” “I feel depressed and irritable when I try to reduce or stop using my phone” and “I don't get enough sleep because too much time is spent on mobile phone.” Items are scored on a 5-point Likert scale (1 = never to 5 = always), with total scores ranging from 13 to 65. In this study, Cronbach's α coefficient was 0.910.

#### FFs and SSBs Consumption Assessment

The self-reported food frequency questionnaire (FFQ) (26) was used to assess the consumption of FFs and SSBs in this study. We measured the five common types of FFs, include Western and Chinese FFs, take-out FFs, pack the food bring back in a plastic bag or plastic lunch box from the school cafeteria or outside. Such as, how many times have you eaten western FFs (KFC, McDonald's, Dicos, etc.) in the last week? In addition, the SSBs also includes five types, which are carbonated drinks (coke, sprite and fanta, etc.), fruit and vegetable juice drinks (e.g., orange), tea drinks (e.g., iced tea), energy drinks (e.g., Red Bull), and dairy beverages (e.g., milk tea). For example, how many bottles of carbonated drinks did you drink every day in the last week? Each question has 4 options (1 = never; 2 = 1–2 times/1 bottle; 3 = 3–4 times/2–3 bottles; 4 = > 5 times/> 4 bottles) were assigned 0 to 3 points. The total score is calculated by adding the scores of the five items, respectively. The higher score being attributed to the higher frequency consumption for FFs and SSBs. In the survey, the Cronbach's α of the FFQ was 0.77.

### Statistical Analysis

The database was entered through Epidata 3.1. Statistical analyses were performed using SPSS Statistics, Version 23.0. Based on participants' responses to the self-harm behavior, individuals who engaged in NSSI and SA in the past year were divided into four groups: NSH (non-self-harm), NSSI (with non-suicidal self-injury exclusively), SA (only with suicide attempts), and NSSI + SA (with both NSSI and SA). Chi square tests were used to evaluate associations between the self-injury group and all demographic factors. Then, Levene's test was used to determine the homogeneity of variance of variables. If the statistics under Levene's test were nonsignificant, one-way ANOVA was adopted; otherwise, a non-parametric Kruskal-Wallis test and pairwise comparisons were utilized. The factors influencing NSSI and SA in this study violated the homogeneity of the variance hypothesis. To reduce the risk of a Type I error, we performed a Bonferroni correction with an adjusted significance level of 0.0083. Finally, to explore the differences of potential influential factors on all levels of self-harm behaviors, we used multivariate logistic regression models. Variables showing a significant difference (*p* < 0.05) between groups were included in the multinomial logistic regression model.

## Results

A total of 14,615 of the 15,486 distributed questionnaires were returned, 645 students refusing to participate. And 14,500 valid questionnaires were included for analysis after excluding those that were incomplete (*n* = 115), which included high levels of missing data (*n* = 79), obvious logical errors (*n* = 20), or inconsistent responses (*n* = 16). The effective response rate was 93.6%. More detailed information on this study has been reported previously (27). The mean age of the participating students was 14.9 years (SD = 1.8), including 7,347 (50.7%) boys and 7,153 (49.3%) girls; 6,881(47.5%) were rural residents, and 7,619 (52.5%) were urban.

### Prevalence and Characteristics of NSSI and SA

Within the sample, NSSI was reported by 27.3% (*n* = 3964) of the students; 4.9% (*n* = 717) reported at least one SA. Participants were divided into the four self-harm groups: NSH, 10,223 students (70.5%); NSSI-only 3,560 (24.6%), SA-only, 313 (2.2%); and NSSI + SA, 404 (2.8%). As shown in Table 1, significant differences were found across the four self-harm groups for age, gender, grade, boarding school, single-child status, residency, family economic status, parents' education levels and number of friends (all *p* < 0.05).

**Table 1:** Demographic characteristic of non-suicidal self-injury and suicide attempt.

**Characteristics**	**Total** **(*N* = 14,500)**	**Self-harm**				***χ*** **^2^/** ***F***
		**NSH** **(*n* = 10,223)**	**NSSI-only** **(*n* = 3,560)**	**SA-only** **(*n* = 313)**	**NSSI+SA** **(*n* = 404)**	
Age (mean, s.d.)	14.86(1.76)	14.95(1.79)	14.58(1.76)	15.27(1.72)	14.75(1.70)	41.86^**^
**Gender**						
Male	7,347(50.7)	5,093(49.8)	1,911(53.7)	164(52.4)	179(44.3)	22.78^**^
Female	7,153(49.3)	5,130(50.2)	1,649(46.3)	149(47.6)	225(55.7)	
**Grade**						
Middle school	7,247(50.0)	4,904(48.0)	1,997(56.1)	140(44.7)	206(51.0)	73.39^**^
High school	7,253(50.0)	5,319(52.0)	1,563(43.9)	173(55.3)	198(49.0)	
**Boarding school**						
Yes	6,830(47.1)	4,947(48.4)	1,586(44.6)	141(45.0)	156(38.6)	28.33^**^
No	7,670(52.9)	5,276(51.6)	1,974(55.4)	172(55.0)	248(61.4)	
**Single child status**						
Only child	4,669(32.2)	3,328(32.6)	1,136(31.9)	100(31.9)	105(26.0)	7.87^*^
Non-only child	9,831(67.8)	6,895(67.4)	2,424(68.1)	213(68.1)	299(74.0)	
**Residency**						
Urban	10,610(73.2)	7,497(73.3)	2,620(73.6)	209(66.8)	284(70.3)	8.69^*^
Rural	3,890(26.8)	2,726(26.7)	940(26.4)	104(33.2)	120(29.7)	
**Family economic status**						
Poor	2,039(14.1)	1,304(12.8)	559(15.7)	71(22.7)	105(26.0)	103.22^**^
Fair	10,010(69.0)	7,226(70.7)	2,379(66.8)	179(57.2)	226(55.9)	
Good	2,451(16.9)	1,693(16.5)	622(17.5)	63(20.1)	73(18.1)	
**Father's education level**						
Primary school	2,195(15.1)	1,469(14.4)	564(15.8)	69(22.0)	93(23.0)	49.18^**^
Middle school	4,706(32.5)	3,278(32.1)	1,203(33.8)	92(29.4)	133(32.9)	
High school	4,120(28.4)	2,977(29.1)	952(26.7)	87(27.8)	104(25.7)	
College or above	3,479(24.0)	2,499(24.4)	841(23.7)	65(20.8)	74(18.3)	
**Mother's education level**						
Primary school	3,315(22.9)	2,272(22.3)	849(23.8)	79(25.2)	115(28.5)	23.73^*^
Middle school	4,664(32.2)	3,265(31.9)	1,178(33.1)	90(28.8)	131(32.4)	
High school	3,786(26.0)	2,754(26.9)	869(24.4)	78(24.9)	85(21.0)	
College or above	2,735(18.9)	1,932(18.9)	664(18.7)	66(21.1)	73(18.1)	
**Number of friends**						
None	437(3.0)	230(2.3)	144(4.1)	32(10.2)	31(7.7)	187.77^**^
1–2	3,099(21.4)	2,044(20.0)	859(24.1)	84(26.8)	112(27.7)	
3–5	6,151(42.4)	4,389(42.9)	1,513(42.5)	109(34.8)	140(34.6)	
≥6	4,813(33.2)	3,560(34.8)	1,044(29.3)	88(28.2)	121(30.0)	

** *p < 0.001,*

* *p < 0.05.*

*n (%)*.

### Four Self-Harm Groups Differences in Influencing Factors

As shown in Table 2, the NSSI+SA group scored highest on all psychological/behavioral problem scales, followed by the SA, NSSI, and NSH groups. The results of the non-parametric Kruskal–Wallis test indicated that the mean (SD) of psychological and behavioral variables significantly different among the four groups of adolescents (all *p* < 0.001), including psychological symptoms, CM, parent-child relationship, PMPU, FFs, and SSBs.

**Table 2:** Means and standard deviations of variables across four self-harm categories.

**Variable**	**NSH**	**Only NSSI**	**Only SA**	**NSSI+SA**	***F***	***Post-hoc*** **comparison**
EPs	26.65 ± 14.68	35.53 ±18.99^a^	43.05 ± 25.70^a^	49.05 ± 24.31	520.37^**^	NSSI+SA>None^c^; Only SA>None^c^; Only NSSI>None^c^; NSSI+SA>Only NSSI^c^; NSSI+SA>Only SA^c^
CPs	12.41 ± 6.98	16.72 ± 9.76^a^	20.46 ± 13.67^a^	23.88 ± 13.68	521.80^**^	NSSI+SA>None^c^; Only SA>None^c^; Only NSSI>None^c^; NSSI+SA>Only NSSI^c^; NSSI+SA>Only SA^c^
SAPs	21.12 ± 12.08	27.48 ± 15.01^a^	32.54 ± 20.32^a^	35.69 ± 18.75	376.27^**^	NSSI+SA>None^c^; Only SA>None^c^; Only NSSI>None^c^; NSSI+SA>Only NSSI^c^; NSSI+SA>Only SA^c^
CM	31.05 ± 10.05	34.57 ± 10.89	44.34 ± 17.38	45.60 ± 14.83	441.60^**^	NSSI+SA>Only SA>Only NSSI>None^c^
FCR	22.53 ± 8.04	24.99 ± 8.69^a^	25.13 ± 8.52^a^	28.13 ± 9.61	130.12^**^	NSSI+SA>None^c^; Only SA>None^c^; Only NSSI>None^c^; NSSI+SA>Only NSSI^c^; NSSI+SA>Only SA^c^
MCR	17.50 ± 8.35	19.31 ± 9.17^a^	20.85 ± 10.35^a^	23.18 ± 10.33	95.88^**^	NSSI+SA>None^c^; Only SA>None^c^; Only NSSI>None^c^; NSSI+SA>Only NSSI^c^; NSSI+SA>Only SA^c^
PMPU	19.15 ± 8.08	21.95 ± 9.51^a^	25.86 ± 14.30^a^	27.66 ± 14.54	232.47^**^	NSSI+SA>None^c^; Only SA>None^c^; Only NSSI>None^c^; NSSI+SA>Only NSSI^c^; NSSI+SA>Only SA^c^
FFs	7.58 ± 2.69	7.89 ± 2.80	9.26 ± 4.10^b^	9.07 ± 3.55^b^	76.11^**^	NSSI+SA>None^c^; Only SA>None^c^; Only NSSI>None^c^; NSSI+SA>Only NSSI^c^; Only SA>Only NSSI
SSBs	6.23 ± 1.81	6.46 ± 1.94	7.82 ± 3.65^b^	7.44 ± 2.98^b^	119.87^**^	NSSI+SA>None^c^; Only SA>None^c^; Only NSSI>None^c^; NSSI+SA>Only NSSI^c^; Only SA>Only NSSI

*A series of non-parametric Kruskal–Wallis tests with a Bonferroni correction of p = 0.0083. Eps, Emotional Problems; CPs, Conduct Problems; SAPs, Social Adaptation Problems; CM, Childhood Maltreatment; FCR, Father-Child Relationship; MCR, Mother-Child Relationship; PMPU, Problematic Mobile Phone Use; FFs, fast foods; SSBs, sugar-sweetened beverages.*

** *p < 0.001.*

a,b *There was no statistically significant difference between groups.*

c *p < 0.0083.*

*Mean (SD)*.

### Pairwise Comparison of Influencing Factors

*Post-hoc* pairwise tests showed that compared with the NSH group, the other three groups scored significantly higher on all study variables. The NSSI + SA group scored significantly higher than the NSSI group. Except for FFs and SSBs, the NSSI+SA group scored significantly higher than the SA-only group on most study variables. The SA-only group and the NSSI-only group showed no significant differences in most study variables except for CM, FFs, and SSBs. In terms of parent-child relationships, the father-child relationship scored higher than the mother-child relationship, and the same trend was seen in the gender subgroup (Table 2).

### The Differences in Influencing Factors Among the Four Self-Harm Groups Were Regulated by Gender

In the gender subgroup, compared with the NSH group, the other three groups scored significantly higher on all study variables. The NSSI + SA group scored significantly higher than the NSSI group. Among males, there was no statistically significant difference in most variables except EPs between the NSSI+SA and SA groups in general (Table 3). Among females, specific differences were analyzed in detail according to all the variables. There was also no statistically significant difference in most variables except CM between the SA group and the NSSI only group. The NSSI+SA group scored significantly higher than the SA group only in the EPs, CPs, SAPs, and FCR variables (Table 4).

**Table 3:** Means and standard deviations of variables across four self-harm categories in male.

**Variable**	**None**	**Only NSSI**	**Only SA**	**NSSI+SA**	***F***	***Post-hoc*** **comparison**
EPs	25.95 ± 14.48	34.91 ± 19.33^a^	42.49 ± 27.23^a^	47.23 ± 25.92	246.09^**^	NSSI+SA>None^c^; Only SA>None^c^;Only NSSI>None^c^; NSSI+SA>Only NSSI^c^; NSSI+SA>Only SA^c^
CPs	12.34 ± 7.05	16.57 ± 10.06^a^	20.62 ± 13.89^a, b^	23.16 ± 14.60^b^	230.83^**^	NSSI+SA>None^c^; Only SA>None^c^;Only NSSI>None^c^; NSSI+SA>Only NSSI^c^
SAPs	21.09 ± 12.31	27.45 ± 15.57^a^	32.96 ± 21.44^a, b^	34.60 ± 19.50^b^	170.27^**^	NSSI+SA>None^c^; Only SA>None^c^;Only NSSI>None^c^; NSSI+SA>Only NSSI^c^
CM	32.04 ± 10.60	35.10 ± 11.48	46.45 ± 18.69^b^	46.77 ± 16.33^b^	197.21^**^	NSSI+SA>None^c^; Only SA>None^c^;Only NSSI>None^c^; NSSI+SA>Only NSSI^c^; Only SA>Only NSSI^c^
FCR	22.37 ± 7.81	24.80 ± 8.56^a^	25.17 ± 7.82^a, b^	27.28 ± 9.11^b^	62.22^**^	NSSI+SA>None^c^; Only SA>None^c^;Only NSSI>None^c^; NSSI+SA>Only NSSI^c^
MCR	17.79 ± 8.55	19.66 ± 9.25^a^	21.27 ± 10.05^a, b^	23.63 ± 9.85^b^	47.62^**^	NSSI+SA>None^c^; Only SA>None^c^;Only NSSI>None^c^; NSSI+SA>Only NSSI^c^
PMPU	19.31 ± 8.45	21.81 ± 9.62^a^	26.66 ± 15.40^a, b^	29.33 ± 16.60^b^	119.12^**^	NSSI+SA>None^c^; Only SA>None^c^;Only NSSI>None^c^; NSSI+SA>Only NSSI^c^
FFs	7.62 ± 2.79	7.90 ± 2.85	9.60 ± 4.43^b^	9.24 ± 3.91^b^	43.33^**^	NSSI+SA>None^c^; Only SA>None^c^; Only NSSI>None^c^; NSSI+SA>Only NSSI^c^; Only SA>Only NSSI
SSBs	6.49 ± 2.11	6.70 ± 2.13	8.80 ± 4.06^b^	8.04 ± 3.59^b^	83.19^**^	NSSI+SA>None^c^; Only SA>None^c^; Only NSSI>None^c^; NSSI+SA>Only NSSI^c^; Only SA>Only NSSI

*A series of non-parametric Kruskal–Wallis tests with a Bonferroni correction of p = 0.0083. Eps, Emotional Problems; CPs, Conduct Problems; SAPs, Social Adaptation Problems; CM, Childhood Maltreatment; FCR, Father-Child Relationship; MCR, Mother-Child Relationship; PMPU, Problematic Mobile Phone Use; FFs, fast foods; SSBs, sugar-sweetened beverages.*

** *p < 0.001.*

a,b *There was no statistically significant difference between groups.*

c *p < 0.0083*.

**Table 4:** Means and standard deviations of variables across four self-harm categoriesin female.

**Variable**	**None**	**Only NSSI**	**Only SA**	**NSSI+SA**	***F***	***Post-hoc*** **comparison**
EPs	27.34 ± 14.84	36.25 ± 18.55^a^	43.66 ± 23.97^a^	50.50 ± 22.91	277.85^**^	NSSI+SA>None^c^; Only SA>None^c^;Only NSSI>None^c^; NSSI+SA>Only NSSI^c^; NSSI+SA>Only SA^c^
CPs	12.47 ± 6.90	16.91 ± 9.40^a^	20.28 ± 13.46^a^	24.45 ± 12.91	295.86^**^	NSSI+SA>None^c^; Only SA>None^c^;Only NSSI>None^c^; NSSI+SA>Only NSSI^c^; NSSI+SA>Only SA^c^
SAPs	21.14 ± 11.84	27.51 ± 14.35^a^	32.07 ± 19.08^a^	36.56 ± 18.14	209.52^**^	NSSI+SA>None^c^; Only SA>None^c^;Only NSSI>None^c^; NSSI+SA>Only NSSI^c^; NSSI+SA>Only SA^c^
CM	30.07 ± 9.38	33.95 ± 10.14	42.01 ± 15.55^b^	44.67 ± 13.49^b^	258.75^**^	NSSI+SA>None^c^; Only SA>None^c^;Only NSSI>None^c^; NSSI+SA>Only NSSI^c^; Only SA>Only NSSI^c^
FCR	22.70 ± 8.26	25.21 ± 8.84^a^	25.09 ± 9.25^a^	28.80 ± 9.96	68.74^**^	NSSI+SA>None^c^; Only SA>None^c^;Only NSSI>None^c^; NSSI+SA>Only NSSI^c^; NSSI+SA>Only SA^c^
MCR	17.22 ± 8.15	18.92 ± 9.06^a^	20.40 ± 10.68^a, b^	3 ± 10.70^b^	48.17^**^	NSSI+SA>None^c^; Only SA>None^c^;Only NSSI>None^c^; NSSI+SA>Only NSSI^c^
PMPU	18.98 ± 7.69	22.11 ± 9.39^a^	24.97 ± 12.99^a, b^	26.34 ± 12.54^b^	118.59^**^	NSSI+SA>None^c^; Only SA>None^c^;Only NSSI>None^c^; NSSI+SA>Only NSSI^c^
FFs	7.55 ± 2.59	7.87 ± 2.73^a^	8.88 ± 3.68^a, b^	8.94 ± 3.25^b^	33.79^**^	NSSI+SA>None^c^; Only SA>None^c^;Only NSSI>None^c^; NSSI+SA>Only NSSI^c^
SSBs	5.96 ± 1.40	6.19 ± 1.65^a^	6.74 ± 2.78^a, b^	6.96 ± 2.29^b^	46.00^**^	NSSI+SA>None^c^; Only SA>None^c^;Only NSSI>None^c^; NSSI+SA>Only NSSI^c^

*A series of non-parametric Kruskal–Wallis tests with a Bonferroni correction of p = 0.0083. Eps, Emotional Problems; CPs, Conduct Problems; SAPs, Social Adaptation Problems; CM, Childhood Maltreatment; FCR, Father-Child Relationship; MCR, Mother-Child Relationship; PMPU, Problematic Mobile Phone Use; FFs, fast foods; SSBs, sugar-sweetened beverages.*

** *p < 0.001.*

a, b *There was no statistically significant difference between groups.*

c *p < 0.0083*.

### Multinomial Logistic Regression

**Figure 1:**
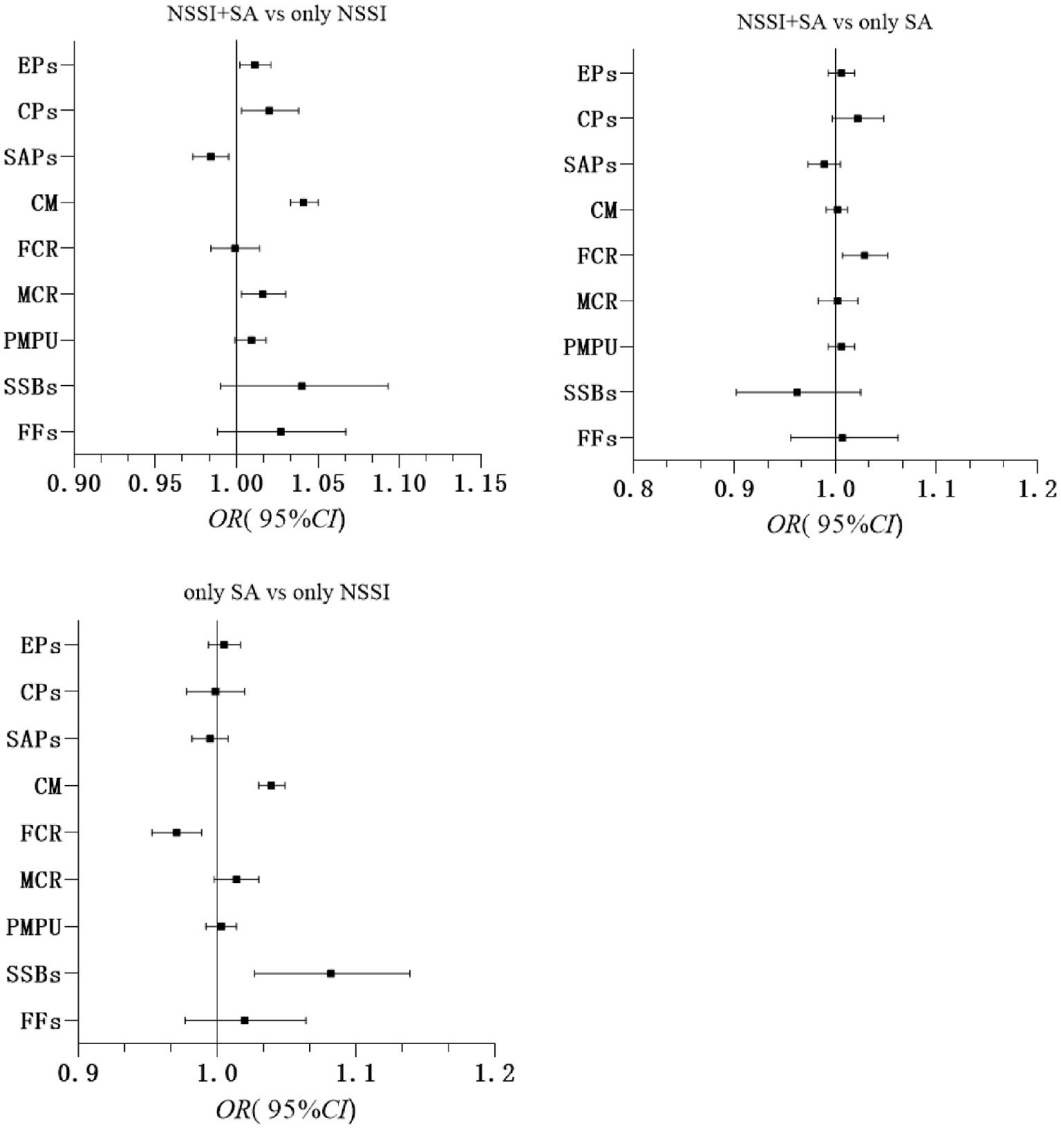
Adjusted odds ratios, 95% confidence intervals from multivariate logistic regressions for self-harm groups. EPs, Emotional Problems; CPs, Conduct Problems; SAPs, Social Adaptation Problems; CM, Childhood Maltreatment; FCR, Father-Child Relationship; MCR, Mother-Child Relationship; PMPU, Problematic Mobile Phone Use; FFs, fast foods; SSBs, sugar-sweetened beverages; Adjusted for age, gender, grade, single child status, boarding school, residency, parents' education levels, family economic status and number of friends. All variables were entered simultaneously, and only the main variables were shown.

In the NSSI+SA group vs. the NSSI group, variables with higher EPs (OR = 1.01, 95%CI: 1.00–1.02, *p* = 0.019), CPs (OR = 1.02, 95%CI: 1.00–1.04, *p* = 0.022), more CM (OR = 1.04, 95%CI: 1.03–1.05, *p* < 0.001), worse MCR (OR = 1.02, 95%CI: 1.00–1.03, *p* = 0.017), and lower SAPs (OR = 0.98, 95%CI: 0.97–0.99, *p* = 0.006) were significant factors of participants with the co-occurrence of NSSI + SA. In the NSSI+SA group vs. the SA group, there was no statistical significance between NSSI+SA and SA-only in the regression model, except for FCR (OR = 1.03, 95%CI: 1.01–1.05, *p* = 0.010). In the SA only group vs. the NSSI group, variables with more CM (OR = 1.04, 95%CI: 1.03–1.05, *p* < 0.001) and SSBs (OR = 1.08, 95%CI: 1.03–1.14, *p* = 0.003) and better FCR (OR = 0.97, 95%CI: 0.95–0.99, *p* = 0.001) were significant factors for differentiating the SA-only group from the NSSI only group (Figure 1).

## Discussion

Our major findings are: (1) more than one in five (27.3%) Chinese adolescents aged 12–18 years reported NSSI, and 4.9% reported having at least once SA; (2) the NSSI + SA group scored the highest on all study variables, followed by the SA-only group, NSSI-only group, and NSH group; (3) In the gender subgroup, compared with the NSH group, the other three groups scored significantly higher on all study variables, and (4) factors that distinguished the NSSI+SA group from the NSSI only group were EPs, CPs, CM, MCR, and SAPs. The only factor that distinguished the NSSI + SA group from the SA group was FCR. Being more CM and SSBs, better FCR were factors differentiating attempted suicide from NSSI.

### Differences Among Four Groups of Adolescents With Self-Harm Behaviors

We found adolescents with either SA or NSSI scored significantly higher than without self-harm adolescents on psychological symptoms and CM, consistent with previous studies (20). Adolescents with SA or NSSI also scored significantly higher than adolescents without self-harm on the FCR, MCR, PMPU, FFs, and SSBs. These findings support the idea that there are significant differences in psychological and behavioral between adolescents with and without self-harm. These results highlight the importance of preventing and treating mental health problems and promoting mental health to prevent self-harm among adolescent.

However, the differences between the SA + NSSI, SA, and NSSI groups in existing studies have not been unified. Our study showed that the NSSI+SA group scored significantly higher on psychological symptoms, CM, parent-child relationship, and PMPU compared with adolescents with SA-only. However, only the differences between SA-only and NSSI-only groups in CM, PPs, and SSBs were significant. Previous studies have reported inconsistent findings. For example, Liang et al. found that there was no significant differences between NSSI + SA and SA only groups (7). The variance of results may be due to differences in the study population and the methods and timing used to assess psychological problems.

Along with previous findings (20, 38), our results indicate that adolescents with SA and NSSI share similar psychological and behavioral characteristics, but these characteristics were more prominent in adolescents in the NSSI + SA group than in the NSSI- and SA-only groups in this study. These findings also support the integrated model theory (8). Studies involving clinical and non-clinical populations have showed that there is a high comorbidity between NSSI and SA, and that individuals who engage in both NSSI and SA have more complex psychopathology, lower psychosocial functioning, and greater aggression and impulsivity than individuals who NSSI alone or SA alone.

Overall, NSSI and SA have different behaviors, but share similar psychological risk characteristics. However, there was no statistically significant differences between SA-only and NSSI-only groups regarding most variables in the total sample of this study.

### Gender Differences

In the gender subgroup, the three groups with self-harm had more psychological and behavioral problems and poorer family relationships than the non-self-harm group overall. However, for these variables, there were no statistically significant differences between the SA + NSSI group and the SA group, except for psychological symptoms and father-child relationship among girls.

A systematic review of the literature showed that higher scores of psychological distress, depression, and female gender were described as major predictors of self-injury in the majority of studies (39). In addition, Jiang et al. (40) showed that among girls, regardless of the level of childhood abuse, low levels of father–child relationships were associated with higher NSSI, while mother–child relationships were not significantly associated with NSSI. Zou et al. (41) also found that poor father–daughter relationships were closely related to girls' health risk behavior, but not statistically significant to boys'. The relationship between father and child is closely related to the occurrence of NSSI in female middle school students.

From what has been discussed above, it may suggest that psychological symptoms and father–child relationships are more sensitive and important in girls than boys. Therefore, special attention should be paid to the cultivation of father–daughter relationships and psychological problems intervention in female students can better prevent the occurrence of self-harm.

### Multinomial Logistic Regression

The findings of our study suggest that adolescents who engage in self-harm behaviors are likely to experience more negative events and have more risk behaviors than those who do not. Compared to the co-occurrence NSSI and SA, individuals with NSSI-only reported fewer psychological symptoms and childhood maltreatment and better mother-child relationship. A prior study revealed that adolescents with both NSSI and SA reported significantly more adverse life events, especially interpersonal negative events, and trauma symptoms than adolescents with NSSI-only (42). A clinical and non-clinical adolescent population study showed that only interpersonal events were associated with both suicidal behaviors and had a moderating effect on the NSSI-suicidal behavior relationship. Nevertheless, patterns of the effects of life events on the NSSI-suicidal behavior relationship did not differ between the two groups (43). Diathesis-stress and kindling effects models suggest various mechanisms from adverse life events (risk-taking behaviors and stressors) increase suicide risk. Risk-taking behaviors may represent a longstanding vulnerability to act impulsively on suicidal thoughts. Stressors may impact the risk of fatal suicidal behaviors in mood-disordered populations (44).

Therefore, in terms of the influencing factors of NSSI and suicidal behaviors, we need to better distinguish stressors and health-risk behaviors and take measures to intervene fundamentally.

### Strengths and Limitations of the Study

The present study balanced the socioeconomic and cultural development of different regions across 4 provinces in China as well as the urban-rural and gender differences. To our knowledge, this was the largest representative, school-based study to compare similarities and differences in multiple domains of behavioral problems, and family environment, and psychological characteristics among adolescents with different types of self-harm in China. This study is comprehensive in that we fully included potential confounding factors such as self-perceived family economic status, number of friends, and parents' education level.

While the findings add to understanding NSSI and SA, there are some limitations. First, the analyses are cross-sectional, so no inferences about causality can be drawn. Prospective cohort studies and intervention studies need to be established for further confirmation. For instance, a New England cohort in adult samples was used to identify factors that distinguish those with different life histories of self-injury conducted by Coppersmith et al. (45). Second, our use of retrospective self-report to collect data may be affected by reporting and recall biases, as well as the existence of sensitive items. These could influence the strength of the observed relationships, and our results may represent a more conservative estimation than is truly present. Third, the list of influencing factors used in our study is reasonably comprehensive but, necessarily, selective. Therefore, we may have missed effects of other important factors of NSSI and SA, such as affective temperaments need to be further studied. A study found that affective temperament-types were independently and more strongly associated with SA than was diagnosis of a major affective disorder in psychiatric inpatients (46). Fourth, although we stratified gender and analyzed the difference of various influencing factors in subgroups, the shortcoming was that we could not estimate the effect size of gender difference in statistical analysis. Finally, this study focused on adolescents in traditional school environments; therefore, the results did not represent adolescents who were absent from school, which is important because studies have shown that adverse experiences and suicidality are more prevalent in individuals with lower educational achievement and socioeconomic status (5). Caution should be exercised when applying the findings to the entire population of adolescents in China.

## Conclusion

In summary, our findings indicate that adolescents with either SA or NSSI have more psychological and behavioral problems and worse family relationships than those without self-harm. Adolescents who engaged in both NSSI and SA in the past year had the most severe psychological and behavioral problems and the most negative family relationships in the total sample. In gender subgroups, this difference between groups may have varied. This study suggests that we should pay more attention to adolescents with more serious psychological and behavioral problems in the future, early identification of risk factors in adolescents' daily life and make efforts to reduce their occurrence, are important implications for protecting adolescents from self-harm behaviors. At the same time, it's also crucial to strength the protective factors, such as improving the quality of parent-child relationship.

## Data Availability Statement

The raw data supporting the conclusions of this article will be made available by the authors, without undue reservation.

## Ethics Statement

The studies involving human participants were reviewed and approved by Ethics Committee of Anhui Medical University (20170290). All study procedures were conducted in accordance with the Declaration of Helsinki. Written informed consent to participate in this study was provided by the participants' legal guardian/next of kin.

## Author Contributions

FT: project administration and supervision. YW: funding acquisition and writing-review. HX: conceptualization and writing-original draft. HX, ZJ, SL, and XZ performed the investigation and formal analysis. SX: data curation. All authors checked interpreted results and approved the final version for publication.

## Funding

This work was supported in part by the National Natural Science Foundation of China (81773453 and 81202223). The funders had no role in study design and collection, analysis, decision to publish, or preparation of the manuscript.

## Conflict of Interest

The authors declare that the research was conducted in the absence of any commercial or financial relationships that could be construed as a potential conflict of interest.

## Publisher's Note

All claims expressed in this article are solely those of the authors and do not necessarily represent those of their affiliated organizations, or those of the publisher, the editors and the reviewers. Any product that may be evaluated in this article, or claim that may be made by its manufacturer, is not guaranteed or endorsed by the publisher.
